# MiR-125a promotes paclitaxel sensitivity in cervical cancer through altering STAT3 expression

**DOI:** 10.1038/oncsis.2016.1

**Published:** 2016-02-15

**Authors:** Z Fan, H Cui, H Yu, Q Ji, L Kang, B Han, J Wang, Q Dong, Y Li, Z Yan, X Yan, X Zhang, Z Lin, Y Hu, S Jiao

**Affiliations:** 1 Department of Oncology, PLA General Hospital, Beijing, China; 2 Department of Oncology, 309th Hospital of PLA, Beijing, China; 3 Department of Orthopedics, PLA General Hospital, Beijing, China; 4 Department of Nuclear Medicine, Peking University First Hospital, Beijing, China; 5 Department of Endocrinology and Metabolism, 264th Hospital of PLA, Shanxi, China

## Abstract

Cervical cancer (CC) is one of the most common malignancies in women. Paclitaxel is the front-line chemotherapeutic agent for treating CC. However, its therapeutic efficacy is limited because of chemoresistance, the mechanism of which remains poorly understood. Here, we used microRNA (miRNA) arrays to compare miRNA expression levels in the CC cell lines, HeLa and CaSki, with their paclitaxel resistance counterparts, HeLa/PR and CaSki/PR. We demonstrate that miR-125a was one of most significantly downregulated miRNAs in paclitaxel-resistant cells, which also acquired cisplatin resistance. And that the upregulation of miR-125a sensitized HeLa/PR and CaSki/PR cells to paclitaxel both *in vitro* and *in vivo* and to cisplatin *in vitro*. Moreover, we determined that miR-125a increased paclitaxel and cisplatin sensitivity by downregulating STAT3. MiR-125a enhanced paclitaxel and cisplatin sensitivity by promoting chemotherapy-induced apoptosis. Clinically, miR-125a expression was associated with an increased responsiveness to paclitaxel combined with cisplatin and a more favorable outcome. These data indicate that miR-125a may be a useful method to enable treatment of chemoresistant CC and may also provide a biomarker for predicting paclitaxel and cisplatin responsiveness in CC.

## Introduction

Cervical cancer (CC) is a common gynecological malignancy that is a leading cause of cancer-related mortality among women worldwide.^1, 2, 3^ Paclitaxel is a front-line chemotherapeutic agent for treating CC, usually in combination with other chemotherapeutic agents.^4, 5^ However, the therapeutic efficacy of paclitaxel is limited, with response rates between 29–63% because of chemoresistance.^4, 5, 6^ Paclitaxel resistance is caused by several mechanisms, including overexpression of P-glycoprotein or other drug efflux pumps,^7, 8^ alterations to microtubules involved in drug-binding or altered expression of tubulin isotypes and microtubule-associated proteins,^9, 10, 11^ alterations to cell cycle and cell survival pathways^12, 13, 14^ and the induction of treatment-related autophagy.^15, 16, 17^ However, the molecular mechanisms by which resistance to paclitaxel occurs are not fully understood and further investigation is required.

MicroRNAs (miRNAs) are a class of endogenous short noncoding RNAs that inhibit post-transcriptional gene expression by binding to target mRNA at their 3′-untranslated region (UTR).^18^ Aberrant expression of miRNA has been associated with cancer chemoresistance, including resistance to paclitaxel.^16, 17, 19^ MiR-125a is an anti-oncogene that has a key role in tumorigenesis in multiple cancers^20^ and is crucial for paclitaxel sensitivity in colon cancer^21^ and cisplatin sensitivity in nasopharyngeal carcinoma.^22^ However, its importance in enabling sensitivity of CC to paclitaxel has not been explored.

In this study, we found miR-125a was significantly downregulated in paclitaxel-resistant CC cells. Overexpressing miR-125a in paclitaxel-resistant cells increases the cell sensitivity not only to paclitaxel both *in vitro* and *in vivo* but also to cisplatin *in vitro* by enabling apoptosis via suppressing STAT3 expression. High expression of miR-125a in CC patients was associated with a favorable response to paclitaxel combined with cisplatin treatment and prognosis. Therefore, upregulating miR-125a may be a novel way to treat chemoresistant CC and miR-125a may be a useful biomarker for predicting the response of CC to paclitaxel and cisplatin.

## Results

### miRNA profiles in paclitaxel-sensitive and -resistant CC cells

**Figure 1:**
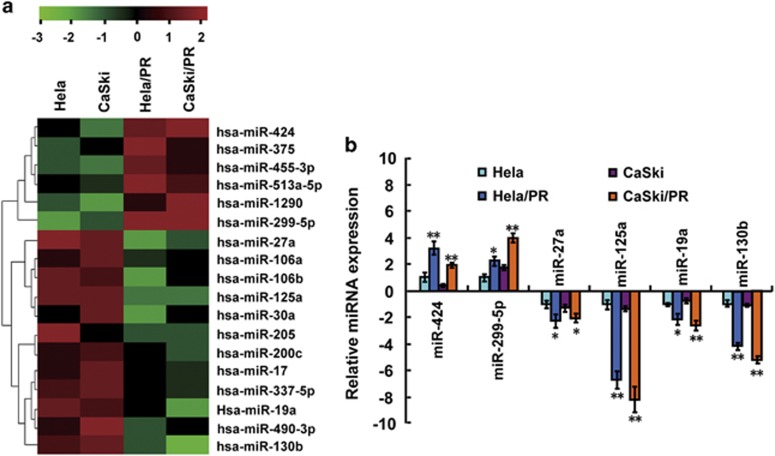
Different expression of miRNAs in paclitaxel-sensitive and -resistant CC cells. (**a**) A heat map showing the miRNA expression profiles and supervised hierarchical clustering analysis for paclitaxel-sensitive (HeLa and CaSki) and -resistant (HeLa/PR and CaSki/PR) CC cell lines. Significantly differentially expressed miRNAs matched the threshold (differential ⩾2-fold, chip signal value >500) and statistical analysis standard were selected (*P*<0.05). Each column represents a cell line and each row shows the relative expression level for individual miRNAs. The red and green colors indicate high of low expression, respectively. (**b**) Expression of six miRNAs (miR-424, miR-229-5p, miR-27a, miR-125a, miR-19a and miR-130b) in the CC cell lines were validated by RT–PCR. *U6* small nuclear RNA was used as an internal control. All values are the mean±s.d. of triplicate measurements, and experiments were repeated three times. *P<0.05, **P<0.01 compared with matched CC cells.

To screen critical miRNAs associated with paclitaxel resistance in CC, we simultaneously analyzed miRNA expression in two CC cell lines (HeLa and CaSki) and their paclitaxel-resistant counterparts (HeLa/PR and CaSki/PR cells; Supplementary Figures 1A and 1B) using miRNA array chips that covered a total 2549 miRNAs. A total of 18 differentially expressed miRNAs were detected in paclitaxel-resistant cells compared with paclitaxel-sensitive cells, including six upregulated miRNAs and 12 downregulated miRNAs (Figure 1a). To further validate the miRNA array chip results, we randomly selected six miRNAs (two upregulated miRNAs, miR-424 and miR-229-5p, and four downregulated miRNAs, miR-27a, miR-125a, miR-19a and miR-130b) to determine their expression by reverse transcription–polymerase chain reaction (RT–PCR). The results were consistent with the miRNA array experiments (Figure 1b). MiR-125a was the most differentially expressed miRNA detected, with more than sixfold lower expression in HeLa/PR and CaSki/PR cells when compared with HeLa and CaSki cells (Figure 1b). As miR-125a expression was associated with paclitaxel-sensitive cells, we hypothesized that miR-125a has a prominent role in paclitaxel resistance of CC.

### Paclitaxel and cisplatin sensitivities are modulated by changes in miR-125a expression *in vitro*

**Figure 2:**
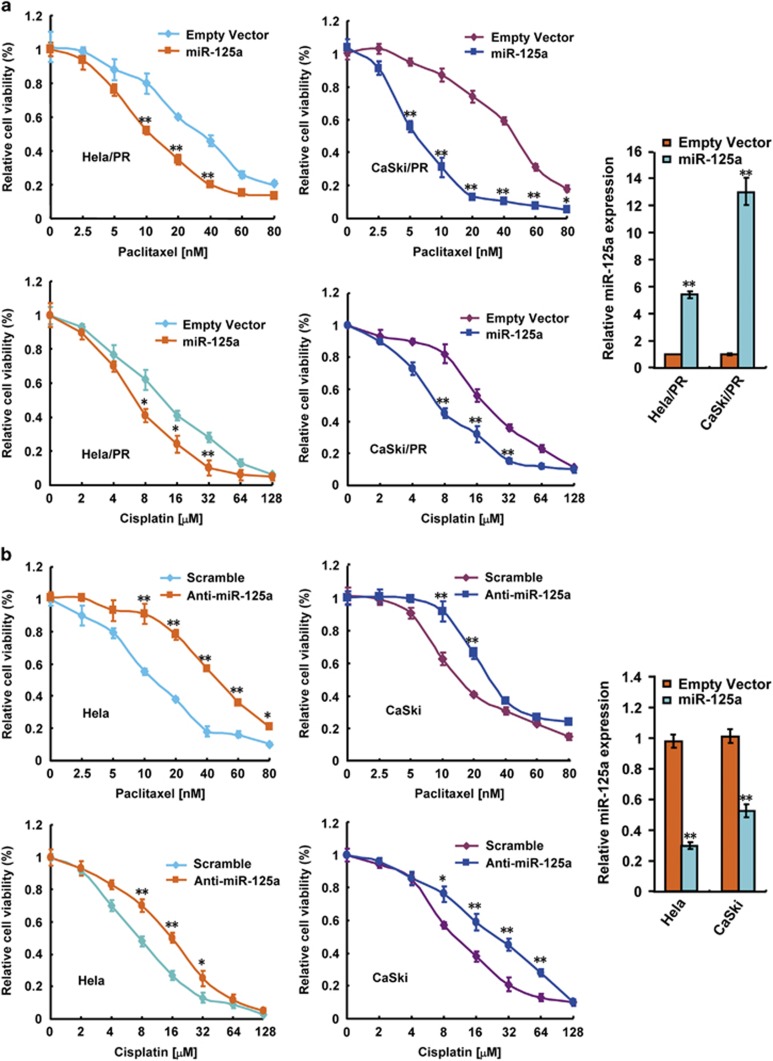
Altered miR-125a expression modulates sensitivity to paclitaxel *in vitro*. (**a**) Overexpression of miR-125a in paclitaxel-resistant cervical cells (HeLa/PR and CaSki/PR) were treated with increasing concentrations of paclitaxel as indicated. After 24 h, cell viability assays were performed using a CCK-8 kit. The group not treated with paclitaxel was presented as 100% viable cells and was used as an internal control for comparison. Histograms on the right show the level of miR-125a overexpression. (**b**) A cell viability assay on paclitaxel-sensitive cells (HeLa and CaSki) following knockdown of miR-125a expression. The cells were treated as in **a**. Histograms on the right show the level of miR-125a knockdown. All values shown are the mean±s.d. of triplicate samples. Experiments were repeated three times (*P<0.05, **P<0.01).

To investigate the biological functions of miR-125a in paclitaxel sensitivity of CC cells, we analyzed the relationship between miR-125a and the IC_50_ of paclitaxel. The IC_50_ values derived from paclitaxel-treated HeLa, CaSki, HeLa/PR and CaSki/PR were 7.76±0.55, 14.1±1.1, 39.89±2.87 and 41.67±3.88 nM, respectively (Supplementary Figure 2A). miR-125a expression was negatively correlated with the IC_50_ values of paclitaxel in all four cell lines (*P*=0.0484, *r*=−0.9372; Supplementary Figure 2B). To confirm the association between paclitaxel resistance and miR-125a expression, miR-125a was transfected into HeLa/PR and CaSki/PR cells, which were then treated with increasing concentrations of paclitaxel. In this viability assay, miR-125a overexpression increased the sensitivity of HeLa/PR and CaSki/PR cells to paclitaxel (Figure 2a). In addition, suppression of miR-125a with specific miR-125a inhibitor in HeLa and CaSki cells increased the paclitaxel resistance (Figure 2b). Recently, Chen *et al.^22^* reported that miR-125a correlated with cisplatin and cisplatin is another important agent for treating CC. Consistent with the results reported by Chen *et al.* in CC, we repeated the experiments as paclitaxel using increasing concentrations of cisplatin. As expected, paclitaxel-resistant cells also acquired cisplatin resistance (Supplementary Figures 1A and 1B). Moreover, similar effects were observed that miR-125a increased cisplatin resistance as paclitaxel (Figures 2a and b). These results suggest that miR-125a expression levels are positively correlated with the sensitivity of CC cells to paclitaxel and cisplatin.

### miR-125a inhibits STAT3 expression by binding to its 3′-UTR

**Figure 3:**
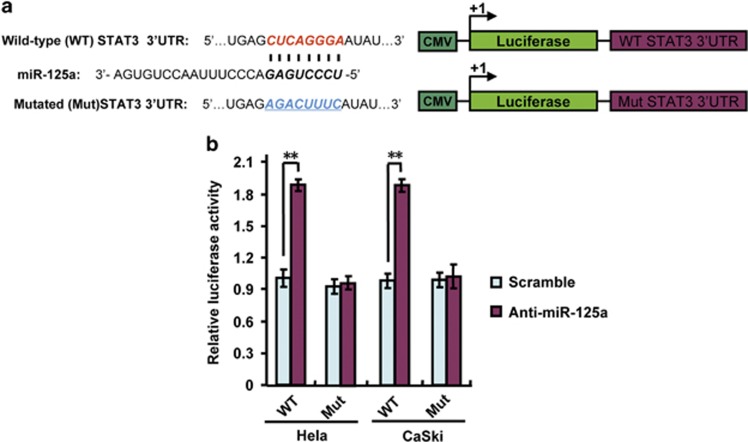
Anti-miR-125a activates STAT3 expression by targeting its 3′-UTR. (**a**) A schematic diagram of the STAT3 3′-UTR luciferase reporter constructs. Bold and italicized fonts indicate putative miR-125a-binding sites in the human STAT3 3′-UTR. Underlining indicates mutations introduced into the STAT3 3′-UTR. (**b**) A miRNA luciferase reporter assay using HeLa and CaSki cells transfected with wild-type or mutated STAT3 reporters plus anti-miR-125a. All values shown are the mean±s.d. of triplicate samples. Experiments were repeated three times (**P<0.01).

Previous studies have indicated that miR-125a directly targets STAT3.^23, 24^ To confirm this, we analyzed the binding ability of anti-miR-125a to wild-type or mutant STAT3 3′-UTR using the luciferase reporter assays. Our results indicated that miR-125a suppression increased wild-type STAT3 3′-UTR reporter activity in HeLa and CaSki cells but did not alter luciferase activity in cells with mutations in the binding sites for miR-125a (Figures 3a and b), which further confirmed previous study results in CC cells. These results indicate that miR-125a inhibits STAT3 expression by directly binding to its 3′-UTR in CC cells.

### miR-125a increased sensitivity of CC to paclitaxel by altering apoptosis because of the downregulation of STAT3

STAT3 has been shown to inhibit apoptosis, the mechanism of which contributes to Lee *et al.*^25^ Thus, we analyzed the expression of STAT3, STAT3 phosphorylated tyrosine 705 (p-STAT3 (Tyr705)), two key apoptosis inhibitors Bcl-2 and Bcl-xL using western blot assays in paclitaxel-sensitive and -resistant CC cell lines. The results indicated that the expression of STAT3 and p-STAT3 (Tyr705) were increased in paclitaxel-resistant cells (HeLa/PR and CaSki/PR) when compared with paclitaxel-sensitive cells (HeLa and CaSki). Similar trends were observed for the expression of Bcl-2 and Bcl-xL, which were also downstream effectors of STAT3 (Supplementary Figure 3).

**Figure 4:**
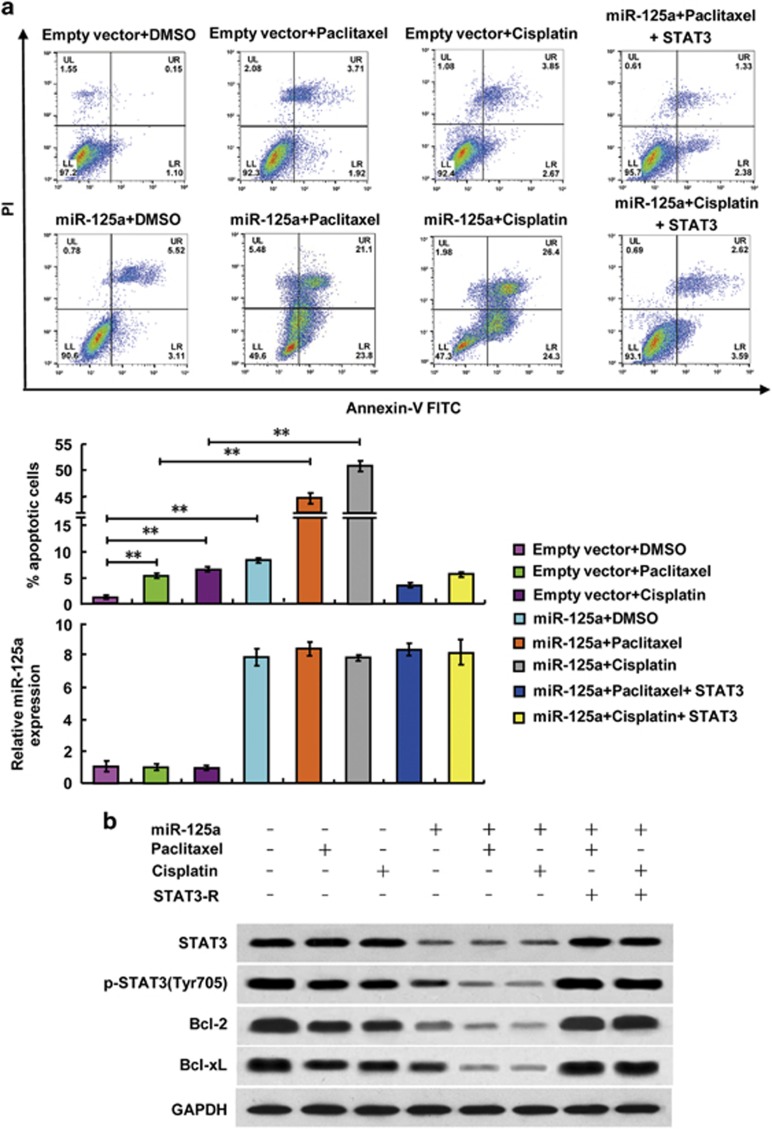
miR-125a suppresses paclitaxel resistance in HeLa/PR cells by downregulating STAT3 resulting in altered expression of apoptosis-related genes. (**a**) Representative flow cytometry analysis of annexin V (1:1000) and propidium iodide (1:1000) staining in HeLa/PR cells transfected with miR-125a or miR-125a plus STAT3 and treated with or without paclitaxel (20 nM) or cisplatin (10 μM) for 24 h. The proportion of apoptotic cells is shown as the mean±s.d. from three independent experiments (**P<0.01). Histograms show the miR-125a expression levels. (**b**) Representative western blots using the indicated antibodies in HeLa/PR cells transfected and treated as in **a**.

As miR-125a directly targeted STAT3 and increased paclitaxel and cisplatin sensitivity, we hypothesized that miR-125a increased paclitaxel and cisplatin sensitivity in CC by enabling apoptosis through downregulation of STAT3. To confirm this hypothesis, we examined the effects of miR-125a and chemotherapeutic agents on the apoptosis of HeLa/PR cells by flow cytometry. The overexpression of miR-125a, paclitaxel treatment and cisplatin treatment, individually, increased the proportion of apoptotic HeLa/PR cells to 8.63%, 5.63% and 6.52%, respectively, compared with that of the control cells (1.25% apoptotic; Figure 4a). Moreover, HeLa/PR cells that overexpressed miR-125a and were treated with paclitaxel or cisplatin had a significantly higher proportion of apoptotic cells (44.9% and 50.7%, respectively; Figure 4a). Similar results were obtained using CaSki/PR cells (Supplementary Figure 4A). Furthermore, treatment with paclitaxel, cisplatin or overexpression of miR-125a inhibited the expression of p-STAT3 (Tyr705), Bcl-2 and Bcl-xL. However, STAT3 expression was suppressed only by miR-125a overexpression. The simultaneous treatment with paclitaxel or cisplatin plus overexpression of miR-125a induced the greatest inhibition of p-STAT3 (Tyr705), Bcl-2 and Bcl-xL (Figure 4b and Supplementary Figure 4B). Inducing the re-expression of STAT3 in the CC cells reversed the effects of miR-125a overexpression and chemotherapeutic agents treatment-induced apoptosis (Figures 4a and b). These data indicate that, miR-125a enables CC cell paclitaxel and cisplatin sensitivity by inducing apoptosis pathway via downregulation of STAT3.

### Role of miR-125a in modulating paclitaxel resistance *in vivo*

**Figure 5:**
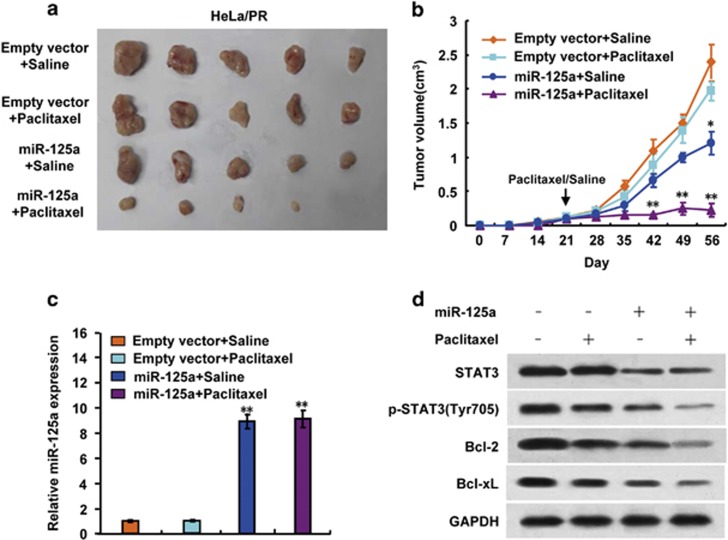
miR-125a mediates paclitaxel sensitivity *in vivo*. HeLa/PR and HeLa/PR cells overexpressing miR-125a were injected into nude mice. After 3 weeks, paclitaxel (15 mg/kg) or saline was intraperitoneally injected once a week for 5 weeks. At the times indicated, tumors were measured with Vernier Calipers (mean±s.d.; *n*=5) (**a**, **b**). Arrow represents the beginning treatment with paclitaxel or saline. (**c**) RT–PCR analysis of miR-125a expression from representative excised tumors. (**d**) Immunoblot analysis of representative excised tumor mass from **a** (*P<0.05, **P<0.01).

After determining that miR-125a mediates paclitaxel resistance in different CC cell lines *in vitro*, we investigated the phenotype of cells overexpressing miR-125a *in vivo*. MiR-125a-overexpressing HeLa/PR cells or control HeLa/PR cells were subcutaneously injected into the backs of BALB/c nu/nu mice. Once the mice developed palpable tumors (>5 mm in diameter within 3 weeks), they were randomly assigned into paclitaxel or saline treatment groups. There was no significant difference in initial tumor volumes (within 3 weeks) between the four groups. Paclitaxel (15 mg/kg) or the same volumes of saline were intraperitoneally injected once a week for 5 weeks. Paclitaxel induced a slight inhibition of tumor growth, however, miR-125a overexpression in paclitaxel-resistant HeLa cells induced an inhibition of tumor growth. The combination of miR-125a overexpression and paclitaxel treatment induced a significant reduction of tumor growth (Figures 5a and b). RT–PCR and immunoblot analysis confirmed that the expression level of miR-125a, STAT3, p-STAT3 (Tyr705), Bcl-2 and Bcl-xL in HeLa/PR cells from the representative tumor mass (Figures 5c and d). These findings indicate that miR-125a upregulation increases paclitaxel sensitivity in paclitaxel-resistant CC cells.

### miR-125a expression correlates with PFS and OS in CC patients who received paclitaxel-based chemotherapy

**Figure 6:**
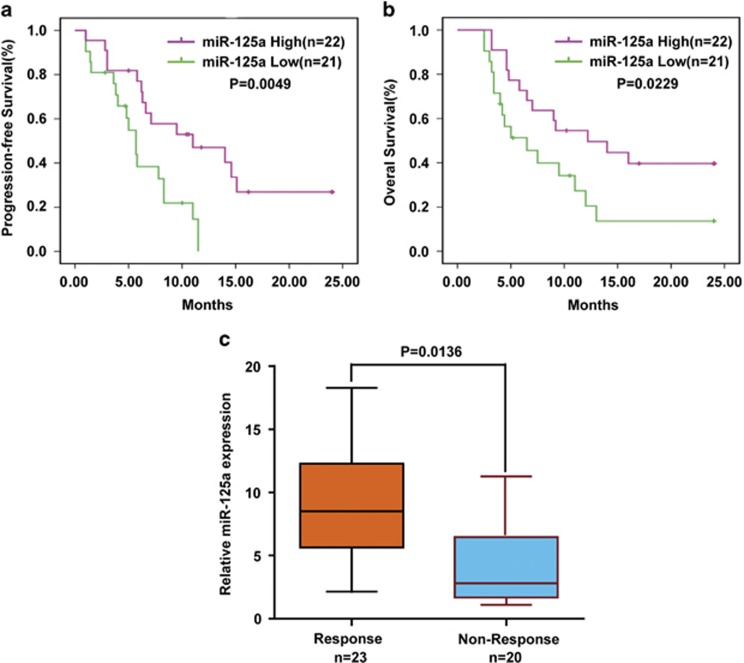
Expression of miR-125a correlates with PFS and OS in paclitaxel treated CC patients. (**a**, **b**) CC patients who received paclitaxel-based chemotherapy were separated into groups based on low or high miR-125a expression levels. Kaplan–Meier survival curves and log-rank tests were used to compare the (**a**) PFS and (**b**) OS between the two groups. (**c**) Expression of miR-125a in patients responded to paclitaxel (*n*=18) and no-responsed (*n*=20) to paclitaxel was compared using the two-tailed Student's *t*-test. U6 small nuclear RNA was used as an internal control.

To study the relationship between miR-125a and paclitaxel resistance in CC, we selected 43 patients who had received paclitaxel combined with cisplatin chemotherapy as the first-line treatment. We stratified patients into high miR-125a expression and low miR-125a expression according to miR-125a expression levels in their CC cells. The effect of chemotherapy on the patient's progression-free survival (PFS) and overall survival (OS) was evaluated according to the Response Evaluation Criteria in Solid Tumors. Patients in the low miR-125a expression group had poorer PFS (*P*=0.0049; Figure 6a), OS (*P*=0.0229; Figure 6b) and response rate (*P*=0.015; Table 1) than those in the high miR-125a expression group. Furthermore, compared with patients who responsed to chemotherapy, miR-125a expression was significantly downregulated in non-responsed patients (*P*=0.0136; Figure 6c). These data indicate important roles for miR-125a in CC paclitaxel resistance.

## Discussion

Chemoresistance in cancers is the main cause of treatment failure.^26, 27^ Although recent data indicate that aberrant miRNA expression is closely linked to chemoresistance by targeting genes related to chemosensitivity^28, 29^ or chemoresistance,^16, 17, 21^ the specific chemoresistance-related miRNAs are largely unknown. More research is needed to identify miRNAs associated with chemoresistance and the mechanisms by which they induce chemoresistance. In this study, we profiled miRNA expression in CC cells by miRNA microarray and compared miRNA expression between paclitaxel-resistant and -sensitive cells. In total, 18 miRNAs had altered expression between the cell lines, several of which have been reported to associate with paclitaxel resistance in CC^28^ and other cancers,^17, 21^ however, miR-125a was first reported in this paper to associate with paclitaxel resistance in CC. miR-125a was the most significantly downregulated miRNA in the resistance cells, and therefore miR-125a may have an essential role in modulating paclitaxel resistance in CC. Based on this, the function of miR-125a in paclitaxel resistance was further analyzed.

In solid tumors, the studies identified miR-125a as an anti-oncogene that inhibits tumorigenesis and cancer progression.^20, 23^ In breast cancer, miR-125a has been demonstrated to inhibit cancer growth and migration by suppressing ERBB2 and ERBB3.^30^ The antitumor functions also confirmed in gastric cancer through ERBB2/miR-125a loop^31^ and by regulating angiogenesis through VEGF-A.^32^ Moreover, Ninio-Many *et al.^33^* indicated miR-125a modulates molecular pathway of motility and migration via Fyn expression in prostate cancer cells. In addition, miR-125a has been associated with many diseases, including autoimmune diseases,^20^ cardiovascular diseases,^34, 35^ microbial infection^36, 37^ and hematological malignancies.^20, 30^ Recent data indicate that miR-125a upregulation sensitized paclitaxel-resistant colon cancer cells to paclitaxel.^21^ However, the association between miR-125a and paclitaxel resistance in CC is unknown. Consistent with the function of miR-125a in colon cancer, we demonstrate that miR-125a expression is suppressed in paclitaxel-resistant CC cells. As expected, overexpressing miR-125a increased the sensitivity of resistant CC cells to paclitaxel and miR-125a knockdown in paclitaxel-sensitive CC cells resulted in resistance to paclitaxel. Meanwhile, we found that miR-125a sensitizes acquired cisplatin resistance of paclitaxel-resistant CC cells. Therefore, a negative correlation between miR-125a expression and chemoresistance was identified in CC.

STAT3 is a well-characterized transcription factor that has been demonstrated to contribute to tumorigenesis and chemoresistance by regulating apoptosis by promoting Bcl-2 and Bcl-xL expression.^25^ In this study, we determined that miR-125a can directly bind to the STAT3 3′-UTR and suppress its expression. Moreover, the expression of STAT3, Bcl-2 and Bcl-xL were significantly increased in paclitaxel-resistant CC cells. Therefore, it is possible that miR-125a regulates chemoresistance by inhibiting STAT3 expression. We determined that enforced overexpression of miR-125a enhanced cell apoptosis, suppressed apoptosis-related proteins. Furthermore, re-expression of STAT3 reversed the function of miR-125a. These data suggest that miR-125a may be a novel molecular mechanism contributing to chemoresistance by inhibiting STAT3 expression in CC. Therefore, miR-125a and STAT3 regulation may be beneficial for the treatment of paclitaxel and cisplatin resistant CC.

Treatment of CC cells with paclitaxel or cisplatin for 24 h did not change the expression of miR-125a or STAT3, but it did decrease p-STAT3 (Tyr705) protein levels. This suggests that paclitaxel and cisplatin inhibits STAT3 not through changing miR-125a/STAT3 pathway but through other pathways, such as the IL-6/Jak2 pathway.^38^ There are several explanations for why miR-125a is downregulated in CC. In this study, we found that overexpressing miR-125a in CC cells and treatment with paclitaxel significantly increased the apoptosis rate than either treatment individually. Thus, sub-populations of cancer cells that have low miR-125a expression may be a potential explanation for resistance against paclitaxel and acquired cisplatin resistance, and combining miR-125a reactivation with paclitaxel and cisplatin treatment may be a useful therapeutic intervention against chemoresistant CC.

In our study, we indicate for the first time that miR-125a upregulation can sensitize HeLa/PR cells to paclitaxel treatment in mice. Tumor volume was more significantly reduced after miR-125a overexpression and paclitaxel treatment than in the other three groups (control group, miR-125a overexpression or paclitaxel treatment groups). We also demonstrated that CC patients with low miR-125a expression had shorter chemotherapy-induced PFS and OS. Moreover, miR-125a expression was significantly downregulated in patients no-responsed to chemotherapy. These data confirm the function of miR-125a in maintaining sensitivity of CC cells to paclitaxel *in vivo* and paclitaxel combined with cisplatin in the clinic.

In conclusion, paclitaxel-resistant CCs have reduced miR-125a expression. MiR-125a negatively regulates paclitaxel and cisplatin resistance in CC by reducing STAT3 expression, which promotes apoptosis and microtubule stabilization. Upregulating miR-125a or inhibiting STAT3 may be useful in combination with paclitaxel and cisplatin for treating chemoresistant CC for these two agents.

## Materials and methods

### Patients and tumor tissues

A total of 43 human CC samples were obtained from the Chinese PLA 309th Hospital and General Hospital with the informed consent of patients and approval for experimentation from the Chinese PLA 309th Hospital and PLA General Hospital. Diagnoses were based on pathological evidence. Patients had not undergone immunotherapy, chemotherapy, hormone therapy or radiotherapy before specimen collection. The clinical stage and histological grades were based on the International Federation of Gynecology and Obstetrics staging. Tissue samples were snap frozen in liquid nitrogen and stored at −80 °C until RNA extraction. All patients underwent intravenous neo-adjuvant chemotherapy (paclitaxel 135 mg/m^2^ and cisplatin 75 mg/m^2^ at 3-week intervals for 5 weeks) after surgery. The effect of chemotherapy was evaluated by following response rate, PFS and OS according to the Response Evaluation Criteria in Solid Tumors.

### Cell culture and transfection

CC cell lines, HeLa and CaSki, were obtained from the American Type Culture Collection (Manassas, VA, USA) and tested for mycoplasma contamination. Paclitaxel-resistant HeLa/PR and CaSki/PR cells were developed from HeLa and CaSki cell lines by treatment with gradually increasing concentrations of paclitaxel in cell culture medium. Briefly, cells were seeded in six-well plates and reached about 80% confluency in fresh medium before treating with paclitaxel. The dose of paclitaxel range from 0.1 to 20 nM and it was increased by a dose gradient that was 25–50% of the previous dose. The next dose was given until the cells were stable in proliferation without significant death.

Stable cell lines overexpressing miR-125a were established by lentiviral transduction using a pCDH plasmid (System Biosciences, Mountain View, CA, USA) carrying miR-125a. All cells were cultured at 37 °C in a humidified atmosphere with 5% CO_2_ in Dulbecco's modified Eagle's medium or RPMI-1640 medium (Life Technologies, Carlsbad, CA, USA) supplemented with 10% fetal bovine serum (Atlanta Biologicals, Lawrenceville, GA, USA) and 1% penicillin/streptomycin (Life Technologies). For transfection,^39^ cells were seeded in 24-well or 6-well plates and then transfected with the indicated plasmids using Lipofectamine 2000 (Invitrogen, Carlsbad, CA, USA) according to the manufacturer's protocol.

### Plasmid construction and reagents

The expression vector for the miR-125a precursor sequence was produced by cloning the PCR product into a pcDNA3.1 vector (Invitrogen) or pCDH plasmid (System Biosciences) with the primers as follows: 5′-CGGGATCCTCTTTCTGTCTCTGGCTCTCAGAA-3′ (forward) and 5′-CGGAATTCAGTGGTCTGGGGTCAGAGGTCA-3′ (reverse). Wild-type and mutated STAT3 3′-UTRs were cloned into a dual-luciferase reporter vector (Promega, Madison, WI, USA) as described previously^39^ using the primers displayed as follows: 5′-CGGAATTCAGGAATCCTGGTCTCAGGACCTC-3′ (forward) and 5′-GCTCTAGATCATACGAGGGCAGACTCAAGT-3′ (reverse) (wild-type STAT3 3′-UTR); 5′-TGGGGCCCCAGCGACGTGTCTGGTTGAGAGACTTTCA-3′ (forward) and 5′-GAAAGTCTCTCAACCAGACACGTCGCTGGGGCCCCA-3′ (reverse) (mutant STAT3 3′UTR). The expression vector for STAT3 was its CDS sequence, which lacks 3′UTR sequence with miR-125a binding sites. The antisense miR-125a oligonucleotide (anti-hsa-miR-125a) and antisense miRNA control were purchased from Qiagen (Valencia, CA, USA).

Anti-Bcl-2 (cat. #sc-429), anti-Bcl-xL (cat. #sc-7195) and anti-GAPDH (cat. #sc-25778) antibodies were purchased from Santa Cruz Biotechnology (Dallas, TX, USA). Anti-STAT3 (cat. #ab126834) and anti-STAT3 (Tyr705) (cat. #ab76315) antibodies were purchased from Abcam (Cambridge, MA, USA). Paclitaxel and cisplatin were obtained from Sigma-Aldrich (St Louis, MO, USA).

### Luciferase reporter assay

Cells were seeded in 24-well plates at a density of 1 × 10^5^ cells per well. The cells were co-transfected with luciferase reporters, either wild-type or mutant STAT3 3′-UTR, in combination with anti-miR-125a or a scramble using Lipofectamine 2000. Forty-eight hours later, cells were harvested and analyzed for luciferase activity using a luciferase assay kit (Promega) according to the manufacturer's protocol.

### miRNA microarray analysis

Total RNA was extracted from HeLa and CaSki cells and their paclitaxel-resistant counterparts using a Qiagen miRNeasy mini kit and following the manufacturer protocol. Total RNA was sent to CapitalBio Corporation (Beijing, China) for miRNA labeling, quality control, chip hybridization and microarray analysis. Briefly, total RNA was labeled with Hy3 and Hy5 fluorescent dyes. Pairs of labeled samples were hybridized to miRCURY LNA miRNA array slides with 2549 human miRNAs. Normalization was performed using a LOWESS filter (Locally Weighted Regression) method to remove system-related variations. An analysis of variance was first applied to produce a miRNA expression profile overview across all samples and then *t*-tests were performed to identify significantly differentiated miRNA expression among all interested combinations of paired groups.

### miRNA extraction and quantitative RT–PCR

Total RNA, including miRNA, was extracted from cultured cells or tissues samples with a miRNeasy Mini kit (Qiagen). Target miRNA was reverse transcribed to complementary DNA using a specific miRNA primer and miScript Reverse Transcription Kit (Qiagen). miRNA expression was measured with a miScript SYBR Green PCR Kit (Qiagen) using the ABI7500 Real-Time PCR System (Applied Biosystems, Foster City, CA, USA). Primers for miRNAs and the endogenous control, U6 gene were displayed in Table 2. The relative fold expression of the target was calculated by the comparative Ct method and was normalized to control.

### Cell viability assay

Cell viability was measured using a CCK-8 Kit (Dojindo, Kumamoto, Japan) according to the manufacturer's protocol. To analyze the effects of miR-125a in combination with paclitaxel or cisplatin, cells transfected with either miR-125a or anti-miR-125a were treated at concentrations of 0, 2.5, 5, 10, 20, 40 and 80 nM with paclitaxel or at concentrations of 0, 2, 4, 8, 16, 32, 64, 128 μM with cisplatin for 24 h. The IC_50_ value was calculated as the concentration of paclitaxel that reduced cell viability by 50%.

### Apoptosis and flow cytometry analysis

Stable miR-125a-overexpressing and miR-125a plus STAT3-overexpressing or empty vector control paclitaxel-resistant cells (1 × 10^6^ cells) were cultured in 60 mm dishes and treated with paclitaxel (20 nM) or cisplatin (10 μM) for 24 h before harvesting. The cells were labeled with propidium iodide and annexin V according to the manufacturer's instructions (BD Biosciences, San Jose, CA, USA). A minimum of 10 000 events for each sample were collected and analyzed using a FACScalibur Flow Cytometer (Becton Dickinson, BD Biosciences).

### *In vivo* cervical tumor xenograft model

All animal experiments were undertaken in accordance with the National Institute of Health Guide for the Care and Use of Laboratory Animals, with the approval of the Scientific Investigation Board of PLA General Hospital, Beijing. Female 6-week-old BALB/c nu/nu mice were purchased from Vital River Inc. (Beijing, China). For the tumor growth model, HeLa/PR cells (2 × 10^7^ cells) stably transfected with the pCDH control vector or pCDH-miR-125a were injected subcutaneously into the backs of BALB/c nu/nu mice (*n*=10), which divided into two groups (*n*=5 based on minimal 30% decrease from 1 g tumors with 250 μg s.d., α error of 0.05 and β error of 0.8) using random number method. After 3 weeks, if the tumor diameter was >5 mm, either paclitaxel (15 mg/kg) or saline was injected intraperitoneally once a week for 5 weeks with no blinding. Tumor sizes were measured at the indicated times using calipers. Tumor volumes were estimated according to the following formula: volume=(longest diameter × shortest diameter^2^)/2.

### Statistical analysis

All *in vitro* experiments were performed in triplicate and repeated three times. Differences between variables were assessed by a *χ*^2^ test or two-tailed Student's *t*-test. The survival rates in relation to miR-125a expression were estimated using the Kaplan–Meier method and the difference in survival curves was analyzed with a log-rank test. The relationship between miR-125a and the IC_50_ of paclitaxel was examined using the Spearman's rank correlation. The SPSS 17.0 statistical software package (SPSS Inc, Chicago, IL, USA) was used to perform all statistical analyses. Data are presented as the means±s.d. *P*<0.05 was considered statistically significant.

## Figures and Tables

**Table 1:** Clinical correlations of miR-125a expression in cervical carcinoma

*Variables*	*miR-125a expression in tumor tissues (T)*		P*-value*
	*High expression*	*Low expression*	
	N*=22 (51.2%)*	N*=21 (48.8%)*	
Age (mean±s.d.), years	53.6±9.78	55.9±10.15	0.911
			
*Tumor size*			
<4 cm	10 (23.3%)	3 (7.0%)	0.045^a^
⩾4 cm	12 (27.9%)	18 (41.9%)	
			
*SCC-Ag*			
<1.5 ng/ml	13 (30.2%)	10 (23.3%)	0.547
⩾1.5 ng/ml	9 (20.9%)	11 (25.6%)	
			
*Histology*			
Adenocarcinoma	2 (4.7%)	3 (7.0%)	0.664
Squamous	20 (46.5%)	18 (41.9%)	
			
*FIGO stage*			
I/II	15 (34.9%)	6 (14.0%)	0.015^a^
III/IV	7 (16.3%)	15 (34.9%)	
			
*Treat with paclitaxel*			
Response	16 (37.2%)	7 (16.3%)	0.015^a^
No response	6 (14.0%)	14 (34.9%)	

Abbreviations: FIGO, International Federation of Gynecology and Obstetrics; SCC-Ag, squamous cell carcinoma antigen. *P*-values of age were calculated by the *t*-test, others by Pearson's chi-square test.

a Statistically significant.

**Table 2:** The real-time PCR primers for miRNAs and U6

*Gene*	*Forward primer*	*Reverse primer*
hsa-miR-424	5′-AGCGGCAGCAGCAATTCATG-3′	5′-GTGCAGGGTCCGAGGT-3′
hsa-miR-299-5p	5′-AGCCGTGGTTTACCGTCCCA-3′	5′-GTGCAGGGTCCGAGGT-3′
hsa-miR-27a	5′-CGCGCTTCACAGTGGCTAAG-3′	5′-GTGCAGGGTCCGAGGT-3′
hsa-miR-125a	5′-CGGCGTCCCTGAGACCCTTT-3′	5′-GTGCAGGGTCCGAGGT-3′
hsa-miR-19a	5′-CGCGGAGTTTTGCATAGTTG-3′	5′-GTGCAGGGTCCGAGGT-3′
hsa-miR-130b	5′-AGCCGACTCTTTCCCTGTTG-3′	5′-GTGCAGGGTCCGAGGT-3′
U6snRNA	5′-TGCGGTGGGTGTCATCAAA-3′	5′-AACGCTTCACGAATTTGCGT-3′

Abbreviation: miRNA, microRNA.
