# Supplementary figures and images for: MiR-125a promotes paclitaxel sensitivity in cervical cancer through altering STAT3 expression
Source: Oncogenesis. 2016 Feb 15;5(2):e197–. doi: 10.1038/oncsis.2016.1 (PMC5154343; doi:10.1038/oncsis.2016.1)

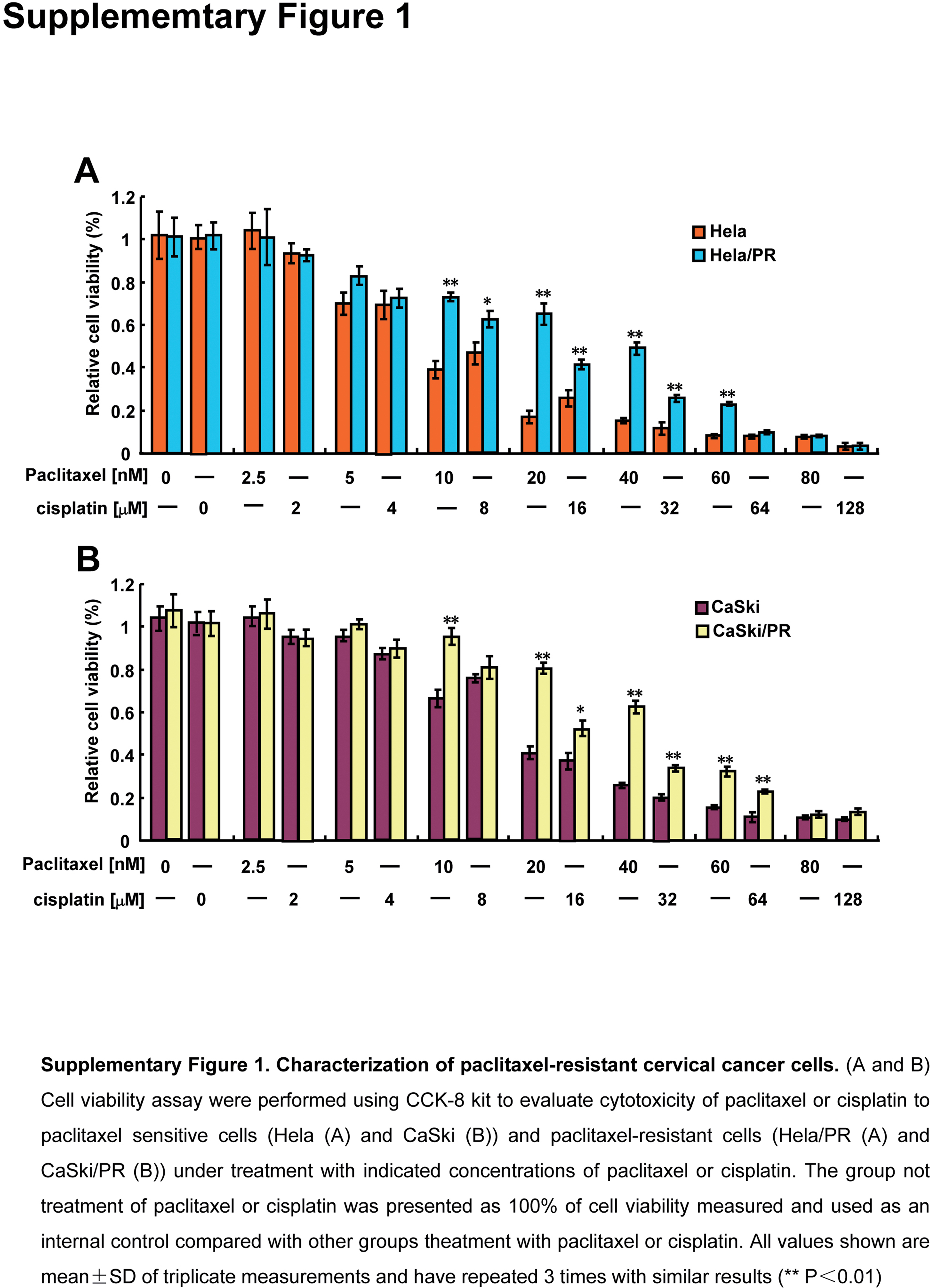

Supplement: Supplementary Figure 1 [file oncsis20161x1.tif]

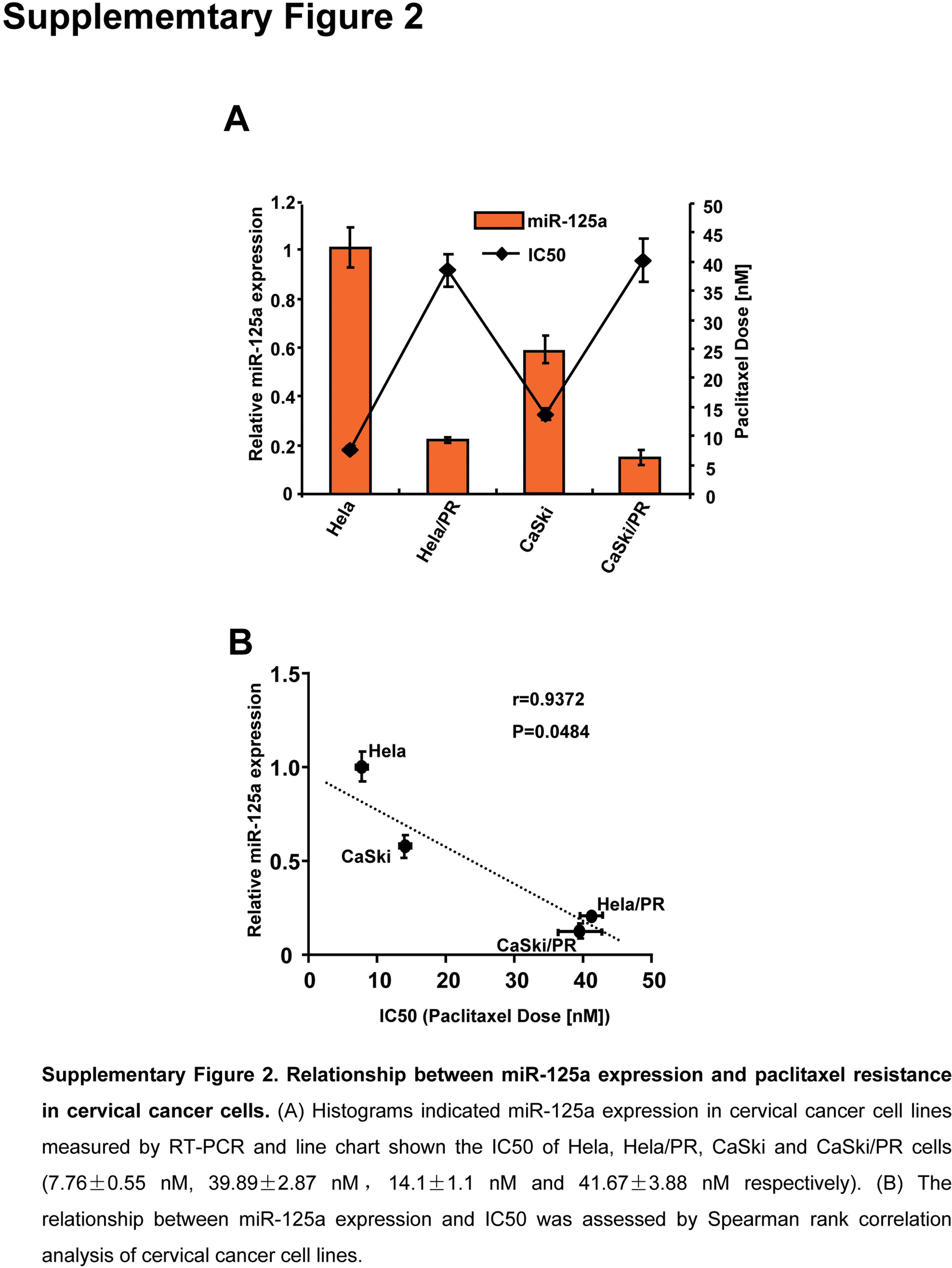

Supplement: Supplementary Figure 2 [file oncsis20161x2.tif]

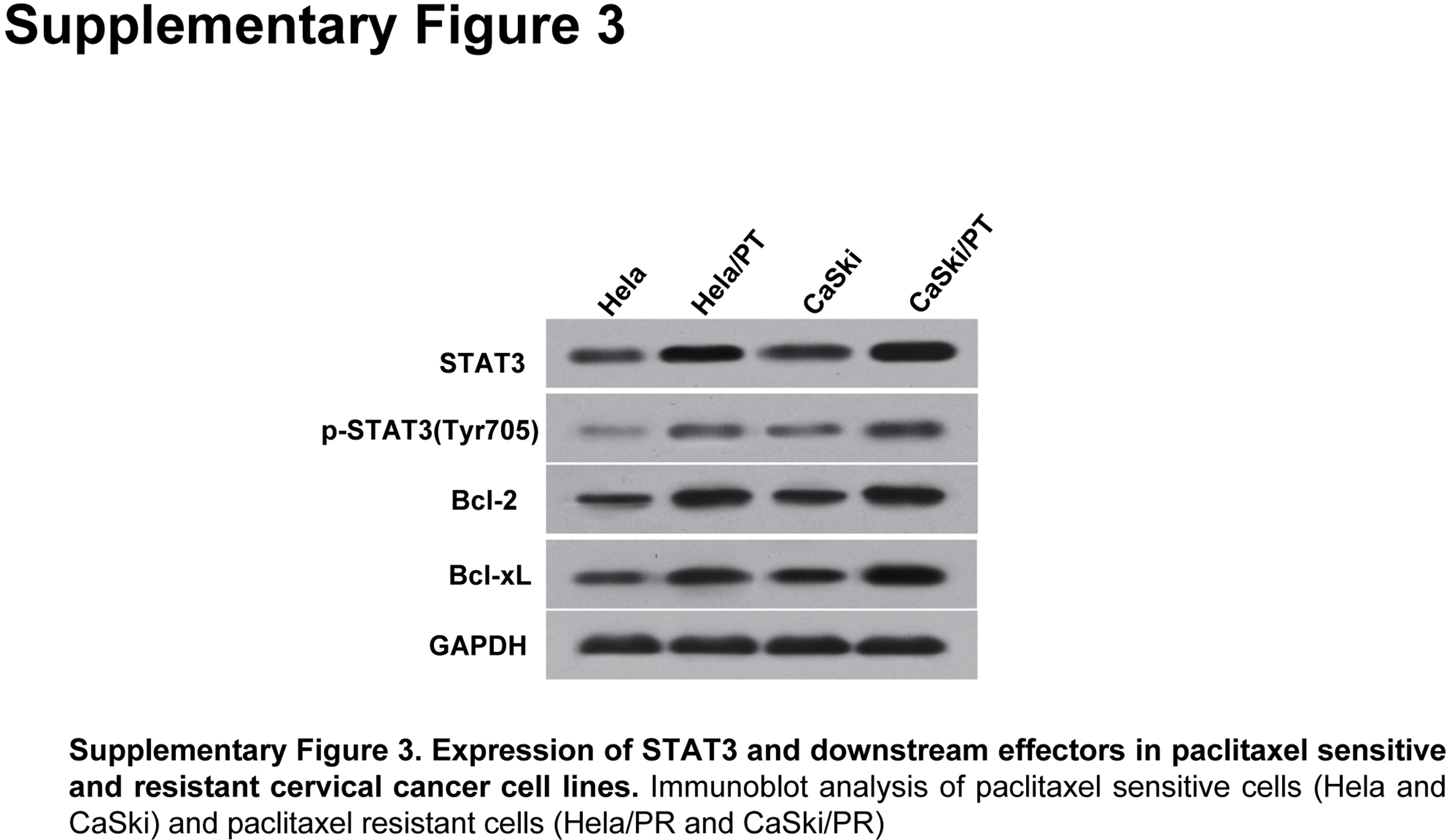

Supplement: Supplementary Figure 3 [file oncsis20161x3.tif]

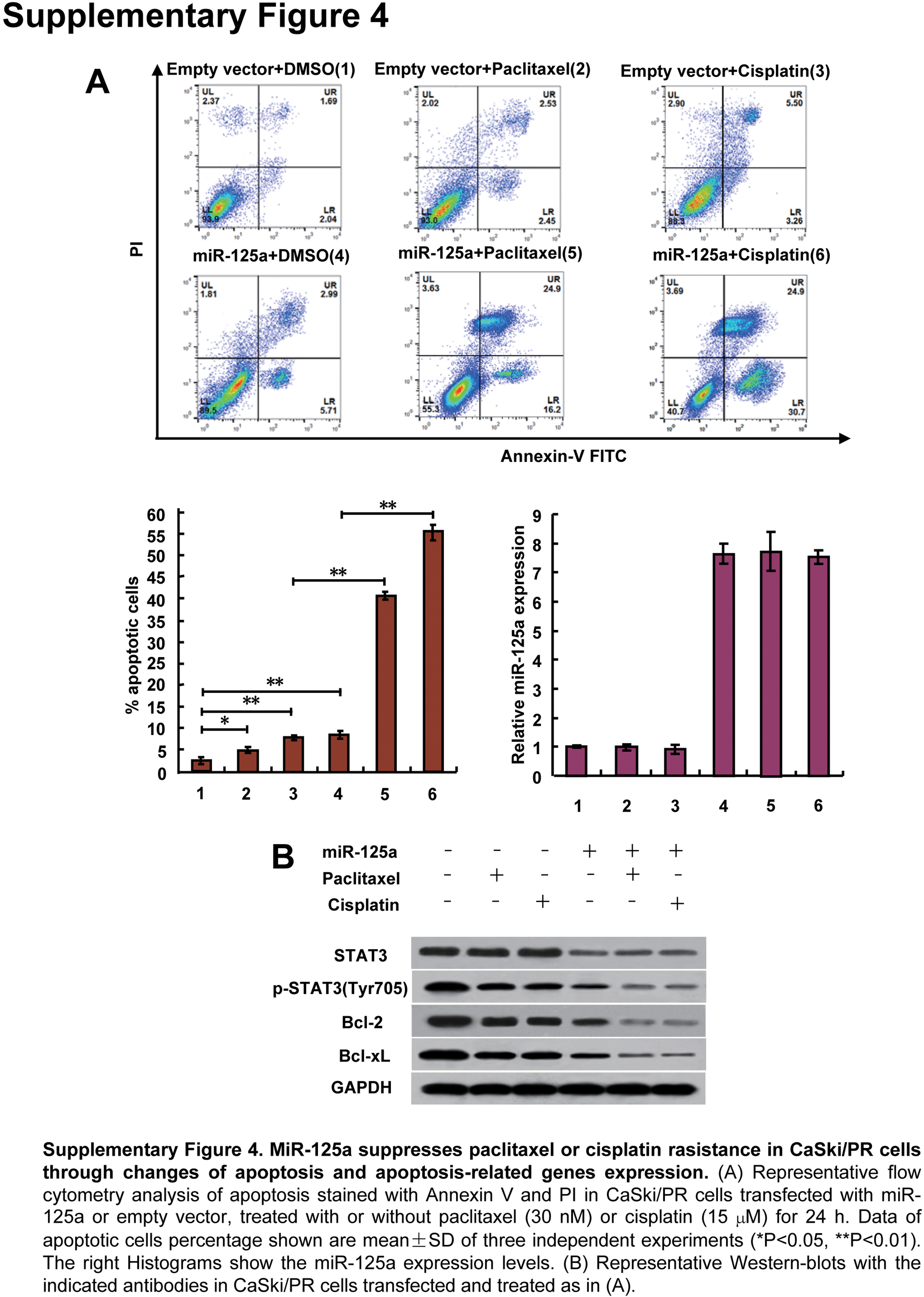

Supplement: Supplementary Figure 4 [file oncsis20161x4.tif]
